# Editorial: World No-Tobacco day: an ethnopharmacological perspective

**DOI:** 10.3389/fphar.2025.1538085

**Published:** 2025-02-12

**Authors:** Arquimedes Gasparotto, Ilana Berlowitz

**Affiliations:** ^1^ Federal University of Grande Dourados, Dourados, Mato Grosso do Sul, Brazil; ^2^ Institute of Complementary and Integrative Medicine, University of Bern, Bern, Switzerland

**Keywords:** tobacco, *Nicotiana*, medicinal plants (herbal drugs), ethnopharmaclogy, Indigenous medicine, nicotine, smoking

While *World No Tobacco Day* primarily focuses on raising awareness about the dangers of tobacco consumption and the tobacco industry, it also represents a unique opportunity for bringing attention to ethnopharmacological perspectives and applications of tobacco (*Nicotiana spp*.), a plant that in Indigenous and traditional medicine systems around the world is considered a medicinal herb, if used accordingly. A recent review reported 108 *N.* species identified to date, the most known ones–*Nicotiana tabacum* L. and *N. rustica* L.– being native to the Americas (Berbeć, 2024). Indeed, millennia-old Indigenous traditions across the Americas have used these plants as medicines and for spiritual purposes (Wilbert, 1993). It was only in the 15th century, after the Spaniards encountered the plant among Indigenous people in South and Central America, that it was brought to Europe (Russell and Rahman, 2015). Eventually, tobacco’s extraction from its Native context, its large-scale commodification and global trade led to today’s addictive and harmful uses and products developed by the transnational tobacco industry. Fortunately, some Indigenous cultures have preserved the knowledge on therapeutic applications of the plant, markedly so in the Amazon (Berlowitz et al., 2024; Berlowitz et al., 2020) but also in other South and Central American regions (Armijos et al., 2014; Groark, 2010) as well as traditional medicine systems around the world that have adopted the plant later on (Binorkar and Jani, 2012).

Nonetheless, recreational uses of tobacco prevail, with worldwide over 1 billion people smoking; although tobacco consumption has decreased in many countries over the last decades (Reitsma et al., 2021), the use of tobacco products among adolescents continues to be a growing concern, especially with the popularization of novel products by the tobacco industry, such as electronic (e−)cigarettes or vaping (Banks et al., 2023). Harmful tobacco consumption is one of the leading risk factors of preventable death worldwide and is associated with increased risk for a range of pathological conditions, including various types of cancer (e.g., lung, larynx), chronic obstructive pulmonary disease, myocardial infarction, stroke, or diabetes (Hajat et al., 2021; West, 2017). The economic and societal costs arising from the tobacco industry and consumption of its products are immense, affecting both individuals, the environment, health systems, and productivity. The World Health Organization (WHO) estimates that tobacco use costs over 1 trillion dollars annually in healthcare expenses and lost productivity (WHO, 2021), which does not yet include the enormous additional costs of tobacco product waste (Lam et al., 2022).

Contemporary treatment approaches for tobacco use disorder or smoking addiction often involve a combination of therapeutic strategies, including cognitive behavioral therapy, nicotine replacement, and in some cases pharmacological treatment with bupropion or varenicline to reduce craving and withdrawal symptoms (Rigotti et al., 2022). Additionally, increasing evidence points to promising ethnopharmacological or integrative therapies employing medicinal plants or other traditional medicine methods (Jang et al., 2016; Lu et al., 2023; Mitra et al., 2023; Tabeshpour et al., 2024). The current Research Topic invited researchers to present ethnopharmacological work on tobacco in traditional medicines, Indigenous uses of tobacco, tobacco in conjunction with transculturation, as well as on harmful uses of tobacco and addiction, associated non-communicable diseases (NCDs), and treatment approaches in this context. While the subsequent contributions indeed speak to several of the above topics, to our regret we did not receive submissions that focused on Indigenous perspectives or traditional medicine applications of the tobacco plant. Interestingly, despite the existence of long-standing medical traditions in this context, and in spite of scientific research on therapeutic potentials of cannabis, psilocybin, and other controversial herbs currently burgeoning (Turek, 2023), tobacco’s clinical applications continue to be a scientifically neglected field. Nonetheless, the current Research Topic offers scientific insight into several important aspects relevant for *World No Tobacco Day*:

In the field of preclinical research, two original studies investigated the use of medicinal plants to address NCDs related to chronic tobacco use; de Medeiros et al. assessed the effects of essential oil from the leaves of *Eugenia pyriformis* Cambess (Myrtaceae) on antimicrobial, nitric oxide production inhibition, and antiproliferative activities in view of treating or preventing damage caused by harmful tobacco consumption. The antiproliferative effects were evaluated against human tumor cell lines, namely breast adenocarcinoma (MCF-7), lung (NCI-H460), cervical (HeLa), and hepatocellular (HepG2) carcinomas. The obtained data demonstrated that the anti-inflammatory, antiproliferative, and antimicrobial activities in the leaves of *E. pyriformis* can add value to the production chain of this plant and open possible avenues for preventing and treating different forms of cancer, including those associated with harmful tobacco use.

A second study focusing on atherosclerosis, another condition for which cigarette smoking is considered a risk factor, examined the use of medicinal plants in this context. More specifically, Cestari et al. investigated the cardioprotective effects of *linia cauliflora* [Mart.] Kausel (Myrtaceae), a very popular fruit species in Brazil. The study aimed to test a preparation obtained from the fruit peels of *P. cauliflora* (EEPC) using male New Zealand rabbits fed for 60 days with a diet enriched with 1% cholesterol. Oral administration of the preparation significantly lowered lipid levels, decreasing systemic oxidative stress and reducing the levels of IL-1b, IL-6, sICAM-1, and sVCAM-1 in the bloodstream. Additionally, a significant reduction of atherosclerotic lesions was observed in all branches of the arteries. The findings suggest that EEPC may be a possible option or complementary approach for addressing the management of atherosclerosis.

Further, Dachen et al. conducted a systematic review and meta-analysis on an important, but still unclear linkage between smoking and cancer treatment success. Recent studies have yielded conflicting evidence regarding the relationship between smoking history and the effectiveness of immune checkpoint inhibitors for advanced lung cancer. While some work found smoking may enhance the response to immunotherapy in patients with lung cancer, other studies could not corroborate these results. Indeed, the current systematic review and meta-analysis concluded that overall, smoking status does not significantly impact the effectiveness of immunotherapy for lung cancer treatment.

Next, assessing a therapeutic approach to treat smoking addiction stemming from Asian traditional medicine, Dai et al. conducted a randomized-controlled double-blind multicenter clinical trial to evaluate the effects of acupuncture on smoking cessation in the presence or absence of nicotine patches. The primary outcome was self-reported smoking abstinence and expiratory carbon monoxide after 8 weeks of treatment. The data showed that acupuncture combined with nicotine replacement therapy was more effective for smoking cessation than acupuncture alone or nicotine replacement patches alone. The study adds further evidence for acupuncture as a promising complementary therapy for smoking cessation, especially if combined with nicotine replacement.

Finally, Zhong et al. investigated knowledge and beliefs around the effectiveness and safety of nicotine, nicotine replacement therapy (NRT), and e-cigarettes used for smoking cessation as held among general practitioners with a special interest (GPwSIs) in respiratory medicine in China. Using a questionnaire they designed for this purpose, the authors conducted a cross-sectional study with a sample of 102 GPwSIs from 21 cities in Sichuan Province, China. The findings showed the physicians possessed varying levels of knowledge, but were generally insufficiently familiar with harms of smoking, e-cigarettes, NRT, and other pharmacological approaches. Further, they commonly overestimated the risks of nicotine, despite its classification as a non-carcinogen. The overall lack of familiarity was attributed to a likely absence of clinical guidelines for primary care practitioners and insufficient training regrading smoking cessation. However, most respondents did not regularly assist patients on this topic. Overall, the study highlights the need for smoking cessation training among GPwSIs in China to improve knowledge and provide better assistance to patients who want to quit smoking.

In conclusion, the Research Topic “World No-Tobacco Day: An Ethnopharmacological Perspective” provides an intriguing set of pre-clinical, clinical, and meta-analytic studies related to harmful tobacco use, associated NCDs and ethnopharmacological or traditional medicine approaches to address these. Collectively, the contributions that comprise the Research Topic present important perspectives for future research and novel therapeutic avenues in conjunction with these topics.
